# Mapping trends and hotspot regarding gut microbiota and host immune response: A bibliometric analysis of global research (2011–2021)

**DOI:** 10.3389/fmicb.2022.932197

**Published:** 2022-07-22

**Authors:** Zhexin Ni, Sheng Wang, Yangshuo Li, Ling Zhou, Dongxia Zhai, Demeng Xia, Chaoqin Yu

**Affiliations:** ^1^Department of Traditional Chinese Gynecology, The First Affiliated Hospital of Naval Medical University, Shanghai, China; ^2^Department of Emergency, The First Affiliated Hospital of Naval Medical University, Shanghai, China; ^3^Department of Trauma Orthopedics, Changhai Hospital, Naval Medical University, Shanghai, China; ^4^Luodian Clinical Drug Research Center, Shanghai Baoshan Luodian Hospital, Shanghai University, Shanghai, China

**Keywords:** gut microbiota, immune response, host, short-chain fatty acid, publications, bibliometrics

## Abstract

**Background:**

Gut microbiota is a complex ecosystem that is vital for the development and function of the immune system, is closely associated with host immunity, and affects human health and disease. Therefore, the current progress and trends in this field must be explored.

**Purpose:**

No bibliometric analysis has been conducted on gut microbiota and host immune response. This study aimed to analyze the current progress and developing trends in this field through bibliometric and visual analysis.

**Methods:**

Global publications on gut microbiota and host immune response from January 2011 to December 2021 were extracted from the Web of Science (WOS) collection database. GraphPad Prism, VOSviewer software, and CiteSpace were employed to perform a bibliometric and visual study.

**Results:**

The number of publications has rapidly increased in the last decade but has declined in the most recent year. The Cooperation network shows that the United States, Harvard Medical School, and Frontiers in Immunology were the most active country, institute, and journal in this field, respectively. Co-occurrence analysis divided all keywords into four clusters: people, animals, cells, and diseases. The latest keyword within all clusters was “COVID,” and the most frequently occurring keyword was “SCFA.”

**Conclusion:**

Gut microbiota and host immune response remain a research hotspot, and their relation to cancer, CNS disorders, and autoimmune disease has been explored. However, additional studies on gut microbiota must be performed, particularly its association with bacterial strain screening and personalized therapy.

## Introduction

As a collection of microorganisms that co-evolve with humans to create an ecological environment and habitat suitable for the host physiology and affect the host phenotype, human microbiota widely exists on the host's skin, mucous membranes, and intestines that are all connected to the external environment (Dominguez-Bello et al., 2019). Therefore, understanding the distribution and function of the microbiota will aid humans in exploring their physiological state. In terms of quantity, most microbiotas have colonized in the intestine, including bacteria, fungi, viruses, archaea, and protozoa, and the majority have not been identified (Aron-Wisnewsky et al., 2012; Lavelle and Sokol, 2020). The complex gut microbiota plays a key role in human health and disease and mainly functions in the digestion of the host, normal metabolism of various substances, supplementation of essential vitamins, identification and resistance to pathogens, maintenance of the functional stability of the intestinal wall barrier, and shaping and regulation of the immune system (Theriot and Young, 2015; Thaiss et al., 2016; Yamamoto and Jørgensen, 2019; Zmora et al., 2019; Chopyk and Grakoui, 2020; Fan and Pedersen, 2021).

The colon is the most voluminous and metabolically active habitat for microbiotas as indicated by the longitudinal increase in species and numbers of gut microbiota from the stomach to the colon (Donaldson et al., 2016). Therefore, the gut microbiota and its metabolites can be easily sampled through the fecal collection and subjected to microbiome profiling and metabolomics for the non-invasive diagnosis of diseases (Ren et al., 2019; Zhang et al., 2020). More than a decade ago, researchers examined the microbiota composition in gut mucosa by fluorescence *in situ* hybridization (FISH) and identified a decrease in *Faecalibacterium prausnitzii* associated with a high risk of postoperative recurrence in ileal Crohn's disease (Sokol et al., 2008). Alterations in the overall structure of the gut microbiota and reduced abundance of the microbiota metabolite butyrate have been found in patients with early diabetes and diabetes (Wu et al., 2020). A fecal sampling of gut microbiota revealed the decreased abundance of *Prevotellaceae* in patients with Parkinson's disease and a positive correlation between the relative abundance of Enterobacteriaceae and the severity of this illness (Scheperjans et al., 2015). These clinical studies indicated that some diseases are associated with alterations in the gut microbiota, and its usage as a biomarker will facilitate and even provide a basis for early diagnosis. Fecal microbiota transplantation (FMT), in which stool from a healthy donor is placed in the gut of another patient, is applied to treat refractory or recurrent Clostridium difficile infection (Gupta and Khanna, 2017; Juul et al., 2018). In a randomized controlled clinical trial, almost one-third of patients with active ulcerative colitis whose disease was effectively relieved by FMT had a Mayo score ≤ 2 (the total Mayo score is a composite of clinical and endoscopic markers and ranges from 0 to 12. 0 = no disease, 12 = most severe disease) (Costello et al., 2019). In addition, FMT can be applied to overcome resistance to antiprogrammed death protein 1 (PD-1) therapy in patients with melanoma or inhibit the decline of endogenous insulin in patients with type 1 diabetes and other diseases (Baruch et al., 2021; Davar et al., 2021; De Groot et al., 2021).

The gut microbiota co-survives with the host through a mutualistic partnership; it can affect the health of the host throughout the life cycle, interact with multiple life activities of the host, and conversely benefit from the host. During the host–microbiota co-evolution, *bifidobacteria*, which are considered probiotics, can be genetically adapted to utilize specific glycans in human secretory fluids (Milani et al., 2017). Neonates have microbiota immediately colonizing their gut after birth partly due to the lactic acid bacteria acquired during delivery or *Bifidobacteria* in the fed breast milk (Dominguez-Bello et al., 2019). Initially, when the immune system of humans is not yet fully developed, a wide variety of microbiotas colonize the host. When the immune system eventually evolves, microbiotas are prohibited from invading the topological interior of the body. The gut microbiota shapes the immune system; the intestinal mucosal layer between the host and the microbiota allows the interaction among host cells, microbiota, and microbial metabolites that produces a range of physiological effects (Rooks and Garrett, 2016). Microbes that penetrate the mucosal layer are engulfed by dendritic cells (DCs) and are actively taken to the mesenteric lymph nodes where they stimulate B cells to secrete immunoglobulin A (IgA). IgA then binds to the gut microbiota after transport across the epithelium to prevent microbial translocation across the epithelial barrier (Macpherson and Uhr, 2004). Thus, the gut microbiota plays an essential role in the formation, regulation, and maintenance of the host immune system, which in turn maintains homeostatic relationships between the internal environment and the gut microbiota. A variety of innate immune receptors are present in the gut epithelium, and the expression of these receptors and signal transduction in response to microbial recognition is crucial to gut homeostasis (Thaiss et al., 2016). Specific deletions in epithelial cells, such as Myeloid differentiation primary response protein 88 (MyD88), a major response protein of myeloid differentiation, disrupt the epithelial barrier and the spatial separation between the mucosal layer constituting the microbiota and the epithelium, ultimately leading to an inflammatory response (Abdel-Gadir et al., 2019).

Interactions between the gut microbiota and immune system have been extensively studied, especially using the popularized methods of 16S rRNA sequencing, metagenomics, and metabolomics that quantitatively and qualitatively analyze the gut microbiota and its metabolism (Bäckhed et al., 2015; Sivan et al., 2015; De Filippis et al., 2016). FISH combined with single-cell imaging, quantitative analysis, metabolic oligosaccharide engineering (MOE), and bio-orthogonal click chemistry (BCC) can assist in visualizing the interaction between the gut microbiota and host in an *in situ* ecological environment (Earle et al., 2015; Geva-Zatorsky et al., 2015). All these techniques contribute to gut microbiota and immune system becoming a research hotspot. However, the large amount of scientific output makes it difficult to accurately locate information and predict hotspots in the future. Therefore, the research trends in gut microbiota–immune system interactions must be summarized and analyzed to provide directions and a basis for future studies.

A bibliometric approach has been adopted for the analysis of the coronavirus disease 2019 (COVID-19) (Xia et al., 2021). However, no studies have focused on the gastrointestinal microbiome, human gastrointestinal microbiome, or gut microbiome in obesity (Yao et al., 2018; Huang et al., 2019; Yuan et al., 2021) and the interaction between gut microbiota and immune system. In this work, bibliometrics and visualization were employed to intuitively and systematically reveal the interactions between gut microbiota and the immune system. Publications and citations, national and affiliated agencies, journal impact, author H-index, and keyword clusters related to the recent decade of gut microbiota and immune system research in the Web of Science (WOS) database were analyzed to evaluate the research hotspot and direction evolution in this field over the past decade and provide new directions for intestinal immunity research (You et al., 2021b). Furthermore, compared to traditional content analysis, bibliometric strategy can provide an objective and holistic overview to discover the spatial and temporal distributions of study status, identify the major and highly-cited scholars, reveal emerging thematic frontiers, and ultimately, contribute to moving the research field forward (You et al., 2021a, 2022).

## Materials and methods

### Data sources and search strategies

**Figure 1:**
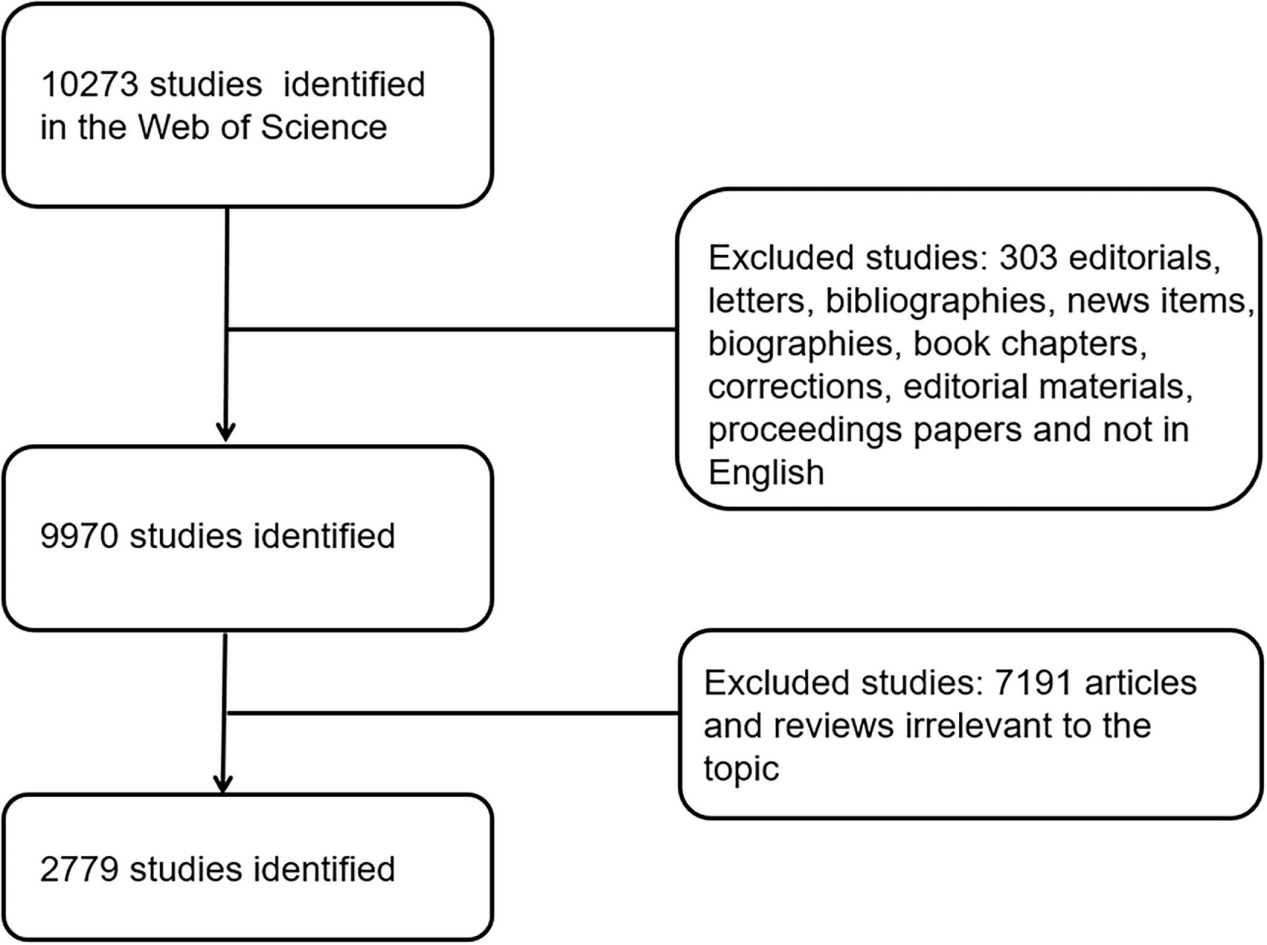
Flow diagram of the inclusion process. The detailed process of screening and enrolment (irrelevant articles were manually screened by two authors through abstracts and full texts, and articles irrelevant to the topic were excluded).

Science Citation Index-Expanded (SCI-E) of Thomson Reuters' WOS is one of the databases covering comprehensive research types and timely data updates. All studies about gut microbiota and host immune response published from 2011 to 2021 were obtained from this database. The search was completed on 31 December 2021, to avoid the daily update of the database that might change the included studies. The following keywords were applied to retrieve all relevant studies: TS [(gut OR intestine OR gastrointestine OR gastro-intestine) AND (microbiota OR microbiome OR flora OR microflora OR bacteria)]AND TS [macrophage OR neutrophil OR (NK cell) OR (natural killer cell) OR (dendritic cell) OR (DC) OR (innate lymphoid cells) OR (ILCs) OR (T cell) OR (T lymphocyte) OR (B cell) OR (B lymphocyte) OR (regulatory T cell) OR (Treg) OR (monocyte) OR immunosuppression OR (immune dysfunction) OR (immune response)] AND Language = English. Only original articles were included, and those irrelevant to the topic were filtered manually. This selection was carried out by two experienced researchers (Z.-X.N. and S.W.) in the field, and disagreements were resolved by an experienced corresponding author (C.-Q.Y.). The article titles and abstracts were first analyzed, and articles that were consistent with the topic content were included based on manual analysis of their content. Full-text articles that could not be judged by the title of the article were downloaded from the Naval Medical University library and further analyzed to ensure that the article topics are relevant to our study. The detailed information on enrolment and selection is shown in Figure 1.

### Data collection and processing

All data (titles, keywords, authors, countries and regions of origin, institutions, published journals, publication dates, H-index, and sum of citations) were extracted from the identified publications by two authors (Z.-X.N. and S.W.). From January 1 2011 until December 31 2021, 10,273 pieces of literature related to the research topic were retrieved by the retrieval formula; 10,054 remained after qualifying the type of literature; and 9,970 remained after qualifying the English language, leaving 2,779 after finally passing the manual screening. GraphPad Prism 8, Microsoft Excel 2016, VOSviewer version 1.6.12, CiteSpace version 5.6. R5 64-bit, and the Online Analysis Platform (http://bibliometric.com/) were applied for presenting, analyzing, and describing the data.

### Bibliometric analysis

Thomson Reuters' WOS studies, especially those focused on biomedicine, were suitable for the article selection. All publications were collected from the website.

The H-index is a useful metric for assessing scientific achievements; this value indicates that a unit has published at least H papers, and each paper has been cited in other publications at least H times (Rad et al., 2010). Relative research interest (RRI) was defined as the number of publications in a particular research field divided by the total number of publications across all fields per year. In this research, the field was gut microbiota and host immune response. Impact factor (IF) was provided by Journal Citation Reports published in 2021. All these factors have a huge contribution to the evaluation of article quality (Eyre-Walker and Stoletzki, 2013), and therefore, were used as key indicators for article evaluation.

VOSviewer, which is a science mapping software tool for constructing and viewing bibliometric maps of countries, journals, or keywords based on co-citation data, was used to analyze different aspects of this research (Yang et al., 2021). As a Java application, CiteSpace can examine and visualize co-citation networks to identify emerging trends in a knowledge domain, research hotspots in a certain field, and future research directions through keyword clustering (Yang et al., 2021).

## Results

**Figure 2:**
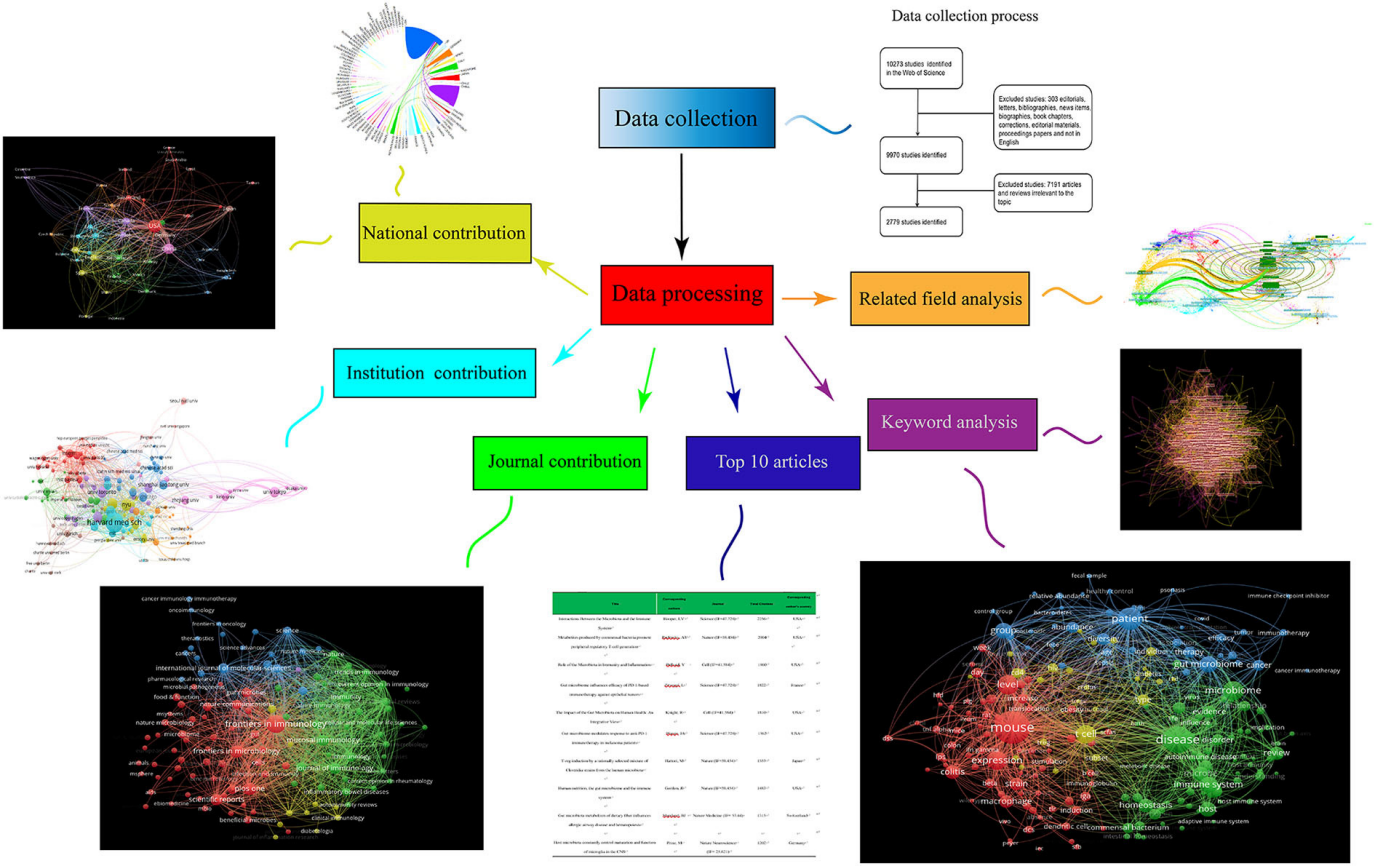
Flow diagram of six aspects of gut microbiota and host immune response. Contributions of countries, contributions of different journals, the top 10 articles, contributions of different institutions, keywords and related fields.

This research focused on six aspects, namely contributions of countries, contributions of different journals, top 10 articles, contributions of different institutions, keywords, and related fields (Figure 2).

### Contributions of countries to global publications

**Figure 3:**
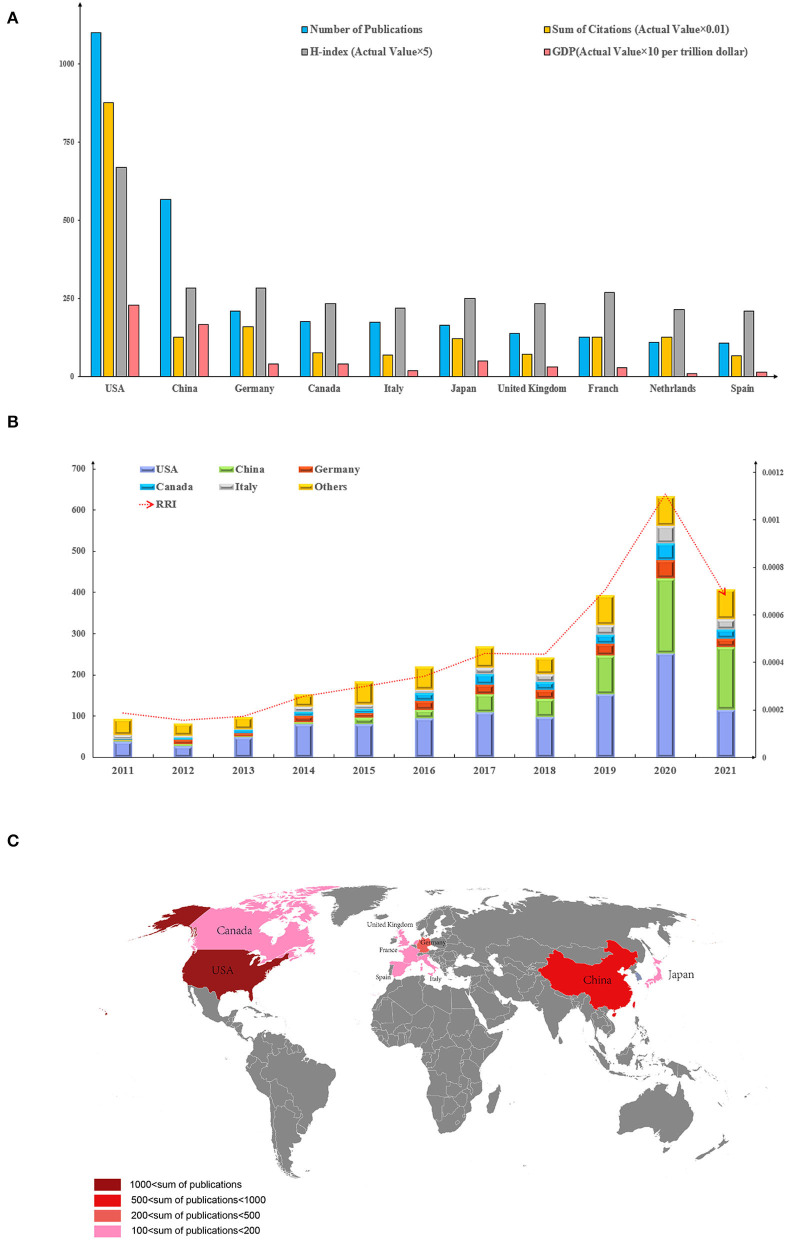
Contributions of different countries/regions to research on gut microbiota and host immune response. **(A)** Number of publications, sum of citations (Actual Value × 0.01), H-index (Actual Value × 5), and GDP (Actual Value × 10 per trillion dollar) in the top 10 countries or regions; **(B)** Number of publications worldwide and the time course of the RRI in gut microbiota and host immune response; and **(C)** Regional clustering characteristics of publications.

The United States ranked first (1,100) in the number of publications between 2011 and 2021, followed by China (568) and Germany (211). The number of publications in a country is positively correlated with its gross domestic product (GDP) (Figure 3A). The number of publications in the United States and China showed a rapid annual increase, especially in 2020. However, when all-field publications were considered, the global interest in the role of gut microbiota in the immune system measured by RRI has steadily increased since 2012 and peaked in 2020 (Figure 3B). In addition, the leading number of publications, the sum of citations, and the H-index of the United States maintained the same advantage. This finding indicates that the publications of the United States had equal qualities and numbers. However, the number of articles published by other countries was not commensurate with their sum of citations and H-index, especially China. The sum of citations in China was 12,832, and that of the United States was 87,736. The H-index of China was 57, and that of the United States was 134. The United States maintained its lead in the number and quality of publications (Figure 3A). Figure 3C shows the regional concentration of research in this field, with Europe, America, and Asia constituting the majority of publications.

**Figure 4:**
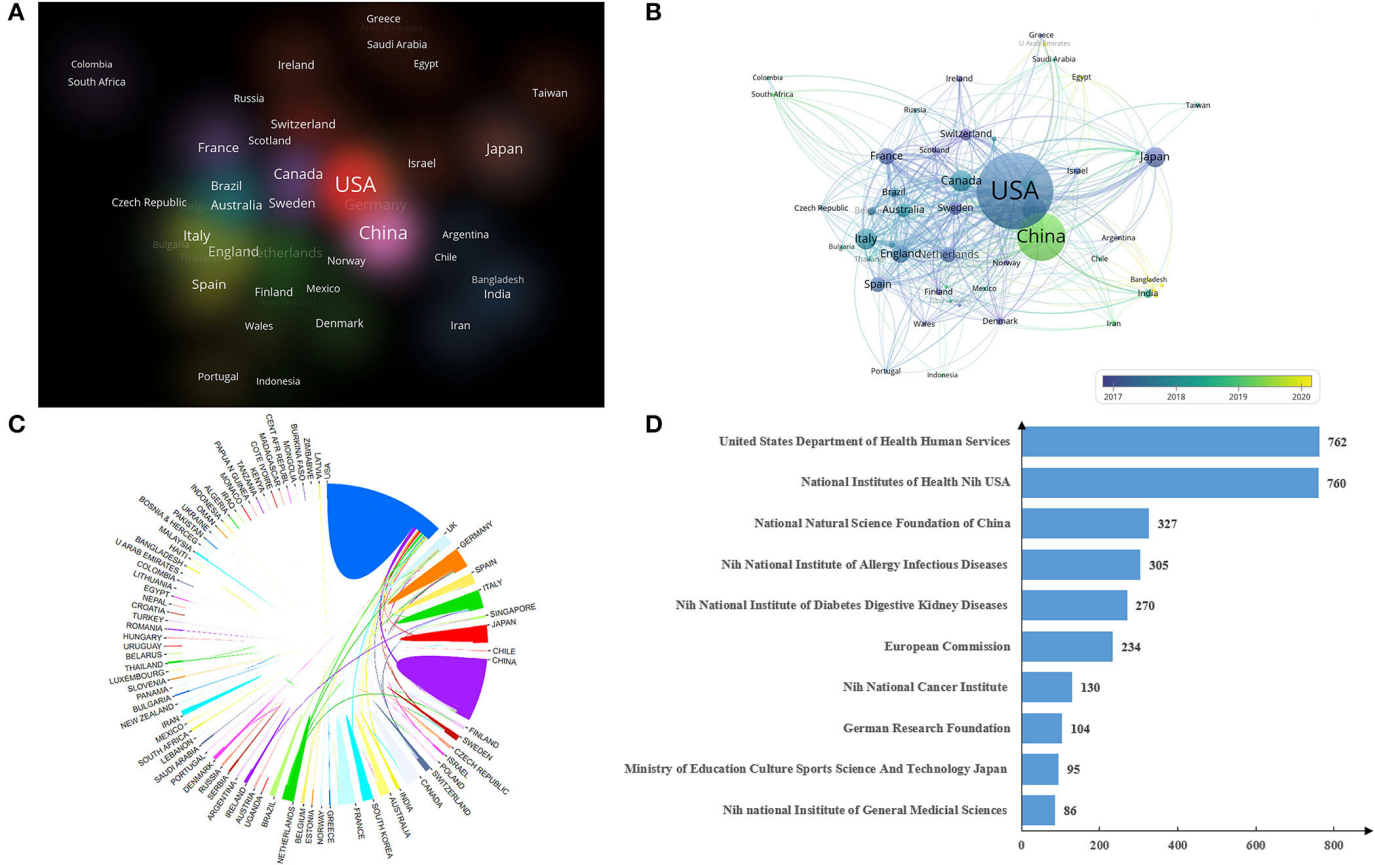
Cooperation network of countries/regions with regard to research gut microbiota and host immune response. **(A,B)** Network of cooperative relations between countries/regions established by VOSviewer. **(C)** Visualized cooperative relations between countries/regions. **(D)** Top 10 funding information.

The number of publications from a specific country is shown in Figure 4A. A large circle indicates a high number of publications. The proportion of latest publications was high, especially in China (Figure 4B). Light color circles represent new publications, and China's research interest in this field has been relatively high in recent years. The cooperation between different countries was also visualized. The United States contributed the most publications and had closer ties to other nations than any other country (Figure 4C). We also summarized the funding information in this field (Figure 4D), and found that the number of projects funded by the United States Department of Health and Human Services was 762, ranking first. The National Institutes of Health Nih United States and the National Natural Science Foundation of China ranked second (760) and third (327).

In Figure 4B, the red color represents the region with a large number of articles published. According to the number of articles published, we find that the Asian region represented by China, Japan, and India has a large number of articles published.

### Contributions of different institutions to publications

**Figure 5:**
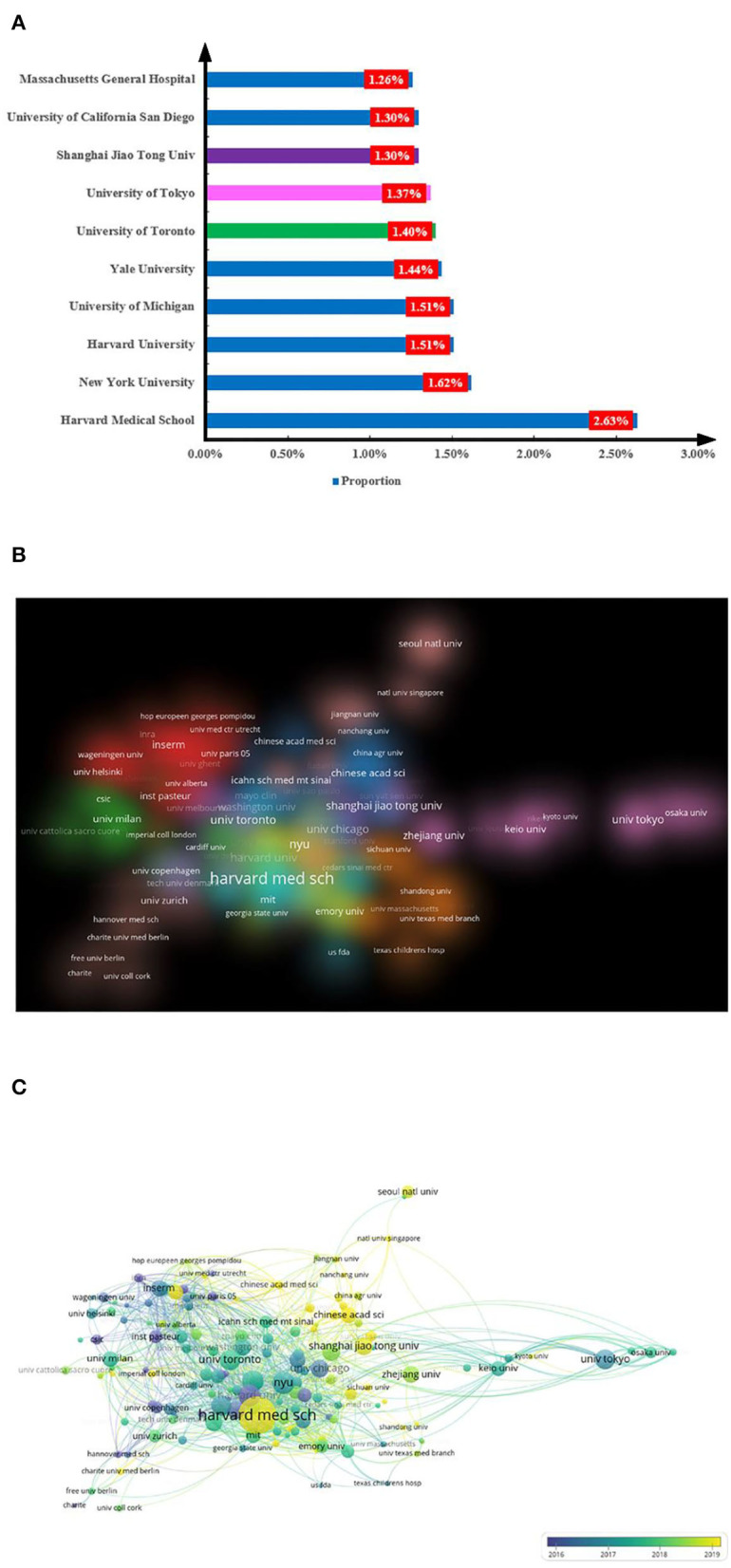
Distribution of institutions engaged in research gut microbiota and host immune response. **(A)** Top 10 institutions by number of publications. Numbers represent the percentage of publications, and different colors represent different countries. **(B,C)** Network of institutions visualized in VOSviewer; the size of circles reveals the number of publications.

The Harvard Medical School in the United States ranked first in the number of publications with 73 records (2.63%), followed by the New York University in the United States with 45 records (1.62%). Harvard University and the University of Michigan in the United States ranked third with 42 records (1.51%). The United States' investment in basic science has recuperated as evidenced by these United States institutions taking up seven positions in the top 10 list, especially the top five. The other three positions were taken by China, Japan, and Canada. This finding shows that the United States has led the advancement of science for nearly a century (Figure 5A).

VOSviewer was used to visualize the cooperation between institutions worldwide. A large circle indicates a higher number of publications (Figure 5B). The institutions with substantial publications were Harvard Medical School, New York University, and Harvard University. This finding is consistent with our previous results. As shown in Figure 5C, yellow represents recent publications and blue represents early publications. Harvard Medical School was the only public institution that had the most publications and had recently focused its attention on the field of gut microbiota and host immune response.

### Contributions of different journals to publications

**Figure 6:**
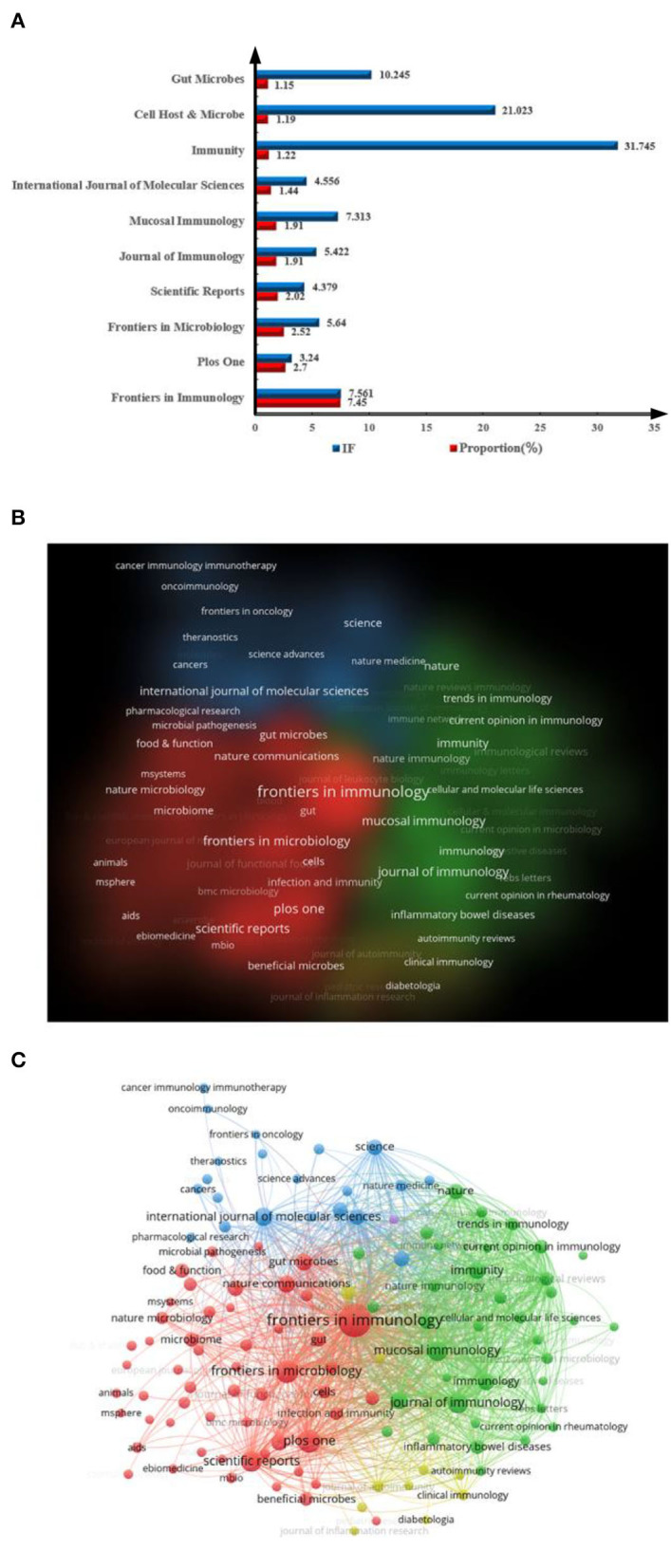
Distribution of journals engaged in research on gut microbiota and host immune response. **(A)** Top 10 journals by number of publications; the blue bar representing IF and red bar representing proportion. **(B,C)** Network of institutions visualized in VOSviewer; the size of circles reveals the H-index.

Figure 6A shows that more than 10% of the relevant papers were published in the top three journals (12.67%). In terms of the number of publications, most papers were published in the *Frontiers in Immunology* (IF= 7.561) with 207 records followed by *Plos One* (IF = 3.24). In terms of IF, the journals which ranked first and second were *Immunity* (IF = 31.745) and *Cell Host & Microbe* (IF= 21.023) with 34 and 33 publications about gut microbiota and host immune response, respectively, and had ranked eighth and ninth in terms of the number of publications, respectively. The top five journals with publication volume had an IF of more than 10 points. A graphic description of journals can be obtained from Figures 6B,C, in which the size of circles reveals the number of publications.

### Top 10 articles

The top 10 articles in the sum of citations are listed in Table 1. “Interactions Between the Microbiota and the Immune System” from the United States was published and cited the most times with 2,256 citations, followed by “Metabolites produced by commensal bacteria promote peripheral regulatory T-cell generation” from the United States with 2,004 citations. The article that ranked third was “Role of the Microbiota in Immunity and Inflammation” from the United States with 1,860 citations. Six of the top 10 articles and all of the top three articles were from the United States. Hence, United States is the leading publisher of high-quality articles. The other top 10 articles were attributed to France, Japan, Germany, and Switzerland. China, the country with the second-highest number of publications, had no contribution to the top 10 high-quality articles. Among the top 10 articles, two focused on PD-1 and two focused on T-cell.

**Table 1:** Top 10 articles in the sum of citations.

**Title**	**Year**	**Corresponding authors**	**Journal**	**Total Citations**	**Corresponding author's country**
Interactions Between the Microbiota and the Immune System	2012	Hooper, LV	Science (IF = 47.728)	2,256	United States
Metabolites produced by commensal bacteria promote peripheral regulatory T-cell generation	2013	Rudensky, AY	Nature (IF = 58.454)	2,004	United States
Role of the Microbiota in Immunity and Inflammation	2014	Belkaid, Y	Cell (IF = 41.584)	1,860	United States
Gut microbiome influences efficacy of PD-1-based immunotherapy against epithelial tumors	2018	Zitvogel, L	Science (IF = 47.728)	1,822	France
The Impact of the Gut Microbiota on Human Health: An Integrative View	2012	Knight, R	Cell (IF = 41.584)	1,810	United States
Gut microbiome modulates response to anti-PD-1 immunotherapy in melanoma patients	2018	Wargo, JA	Science (IF = 47.728)	1,562	United States
T-reg induction by a rationally selected mixture of Clostridia strains from the human microbiota	2013	Hattori, M	Nature (IF = 58.454)	1,553	Japan
Human nutrition, the gut microbiome and the immune system	2011	Gordon, JI	Nature (IF = 58.454)	1,483	United States
Gut microbiota metabolism of dietary fiber influences allergic airway disease and hematopoiesis	2014	Marsland, BJ	Nature Medicine (IF = 53.44)	1,315	Switzerland
Host microbiota constantly control maturation and function of microglia in the CNS	2015	Prinz, M	Nature Neuroscience (IF = 25.621)	1,202	Germany

### Analysis of keywords in publications about gut microbiota and host immune response

**Figure 7:**
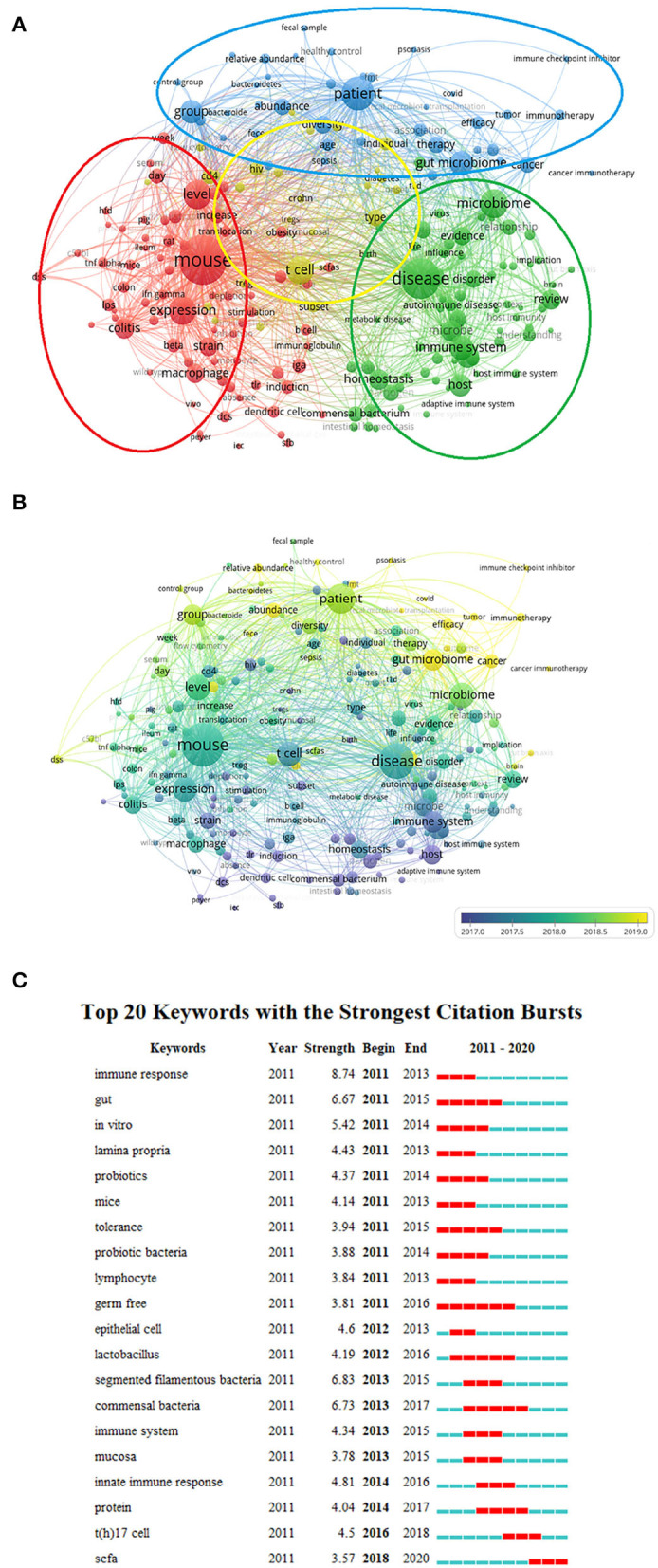
Co-occurrence analysis of all keywords in the publications on gut microbiota and host immune response: **(A)** Mapping of the keywords in the field of gut microbiota and host immune response. The size of the circle represents the frequency of keywords, and different colors represent different clusters. **(B)** Distribution of keywords according to the average time of appearance; blue represents an early appearance, and yellow represents a late appearance. **(C)** Top 20 keywords cited the most frequently from 2011 to 2020 and have received continuous attention for a period of time. The red bars represent frequently cited keywords during this time period, and the green bars represent infrequently cited keywords.

The keywords extracted from 2,779 publications were analyzed using VOSviewer. Keywords were defined as terms that were frequently mentioned and occurred more than 60 times within titles and abstracts in all papers during analysis. Figure 7A shows that among the 213 keywords, the top three were mouse (3,242 times), disease (2,261 times), and patient (1,617 times). Detailed data on the co-occurrence of all included keywords are presented in Figure 7A. Compared with the keywords that appear most frequently, recent keywords represent current research hotspots.

Different colored circles also represent the four clusters: the blue cluster represents people, the red cluster represents animals, the yellow cluster represents cells, and the green cluster represents diseases.

As shown in Figure 7B, based on the different average appearing year (AAY) of keywords, the VOSviewer marked keywords are included in the map with different colors. Specifically, blue indicates that the word appeared relatively early and yellow indicates a recent appearance. After keyword processing, the most frequently mentioned keywords within the cluster of people were COVID (60 times), immune checkpoint inhibitor (67 times), and immunotherapy (184 times). Within the cluster of animals, SCFA (short-chain fatty acid, 184 times) had the highest frequency of occurrences, followed by supplementation (250 times) and dss (179 times). Within the cluster of cells, the primary keywords were mait cell (80 times), percentage (75 times), and flow cytometry (121 times). Within the cluster of diseases, the primary keywords were gut brain axis (91 times), metabolite (603 times), and brain (93 times). The latest keyword within all clusters was COVID with the latest AAY of 2020.8833. Additional details on keywords are presented in Supplemental Table 1.

Among all 213 keywords, 20 had lasted for the longest time (Figure 7C). The newest salient keyword was “SCFA,” which was highlighted from 2018 to 2020 for ~3 years. “Germ free” lasted the longest (6 years) from 2011 to 2016, indicating that this term has been the focus of previous research. Since 2017, “SCFA” has gradually become the focus of research in this field. Similarly, “SCFA” cluster of animals was also the latest keyword.

### A dual-map overlay analysis of gut microbiota and host immune response

**Figure 8:**
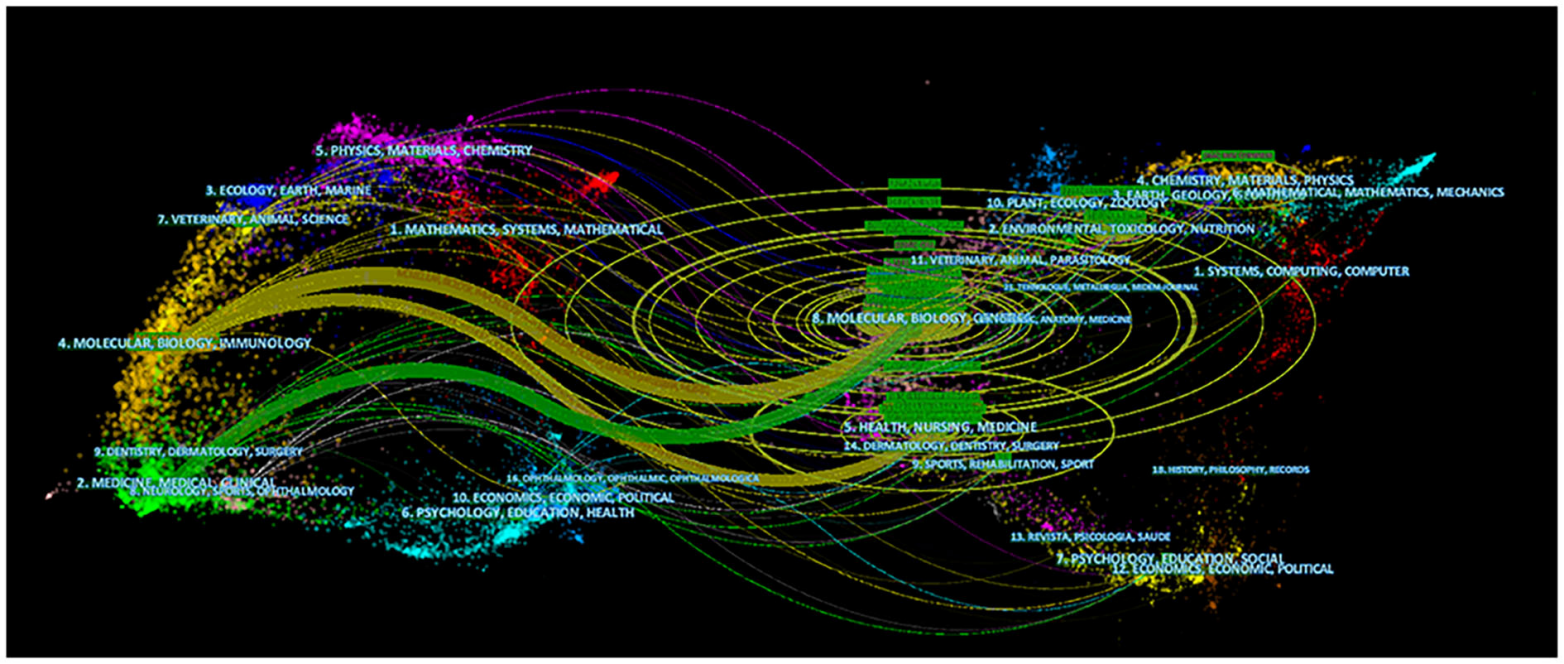
A dual-map overlay analysis in gut microbiota and host immune response. The left side represents the fields of articles included in the study, and the right side represents the fields of references of articles.

As shown in Figure 8, the 2,779 included articles were mainly divided into three fields: the first included medicine, medical, and clinical; the second included molecular, biology, and immunology; and the third included neurology, sports, and ophthalmology. In addition, the references of these 2,779 articles were mainly distributed in the following four fields: the first included health, nursing, and medicine; the second included sports and rehabilitation; the third included health, nursing, and medicine; and the fourth was molecular biology genetics.

In summary, the field of gut microbiota and host immune response mainly involved subdisciplines in clinical medicine and molecular biology.

## Discussion

### Research trends in gut microbiota and host immune response

Although many gut microbiota-related bibliometric studies have been published (Huang et al., 2019; Zhu et al., 2020, 2021; Yuan et al., 2021), no bibliometric study has been conducted on the relationship between gut microbiota and immune system. To the authors' knowledge, this report is the first to use bibliometric and visual analysis for immune research in the gut microbiota field. Important publication information such as country, year, institution, and keywords was analyzed by visualization methods such as GraphPad Prism, VOSviewer, and CiteSpace.

Since 2011, the number of related publications showed an overall increasing annual trend, but the number dropped back in 2021 (Figure 3B). With advances in genomic technologies and the widespread use of FISH, MOE, and BCC research methods, this field has gradually become popular among researchers. The accelerating growth in the number of publications from 2013 benefited from the publication of two articles on the impact of gut microbiota on regulatory T (Treg) cells (Arpaia et al., 2013; Atarashi et al., 2013). This phenomenon has paved the way for gut microbiota research. Furthermore, the rise was accelerated further between 2018 and 2020 due to the publication of two articles in 2018 on basic and clinical research on the effects of gut microbiota on PD-1 therapy (Gopalakrishnan et al., 2018; Routy et al., 2018). All these articles appeared in the TOP 10 (Table 1), indicating that research between the gut microbiota and the host immune response has received widespread attention and holds promise as a possible cure for cancer. However, a decrease in research interest occurred in 2021. The involvement of experts in other fields in this area and a refined research strategy and methodology are needed to break through the research hurdles that have recently emerged. In terms of global distribution (Figure 3C), publications have been produced longitudinally from the east and west hemispheres. This trend has facilitated studies of the impact of the gut microbiota on the immune system in populations with different geographical and dietary differences.

The United States has an absolute advantage in the study of immunity and gut microbiota as reflected by its largest number of publications, citation impact, and H-index ((Figure 3A). GDP is an important factor in the number of publications of a country. Among the 10 institutions that contributed the most to publications in the field, the top five were all institutions affiliated with the United States, with Harvard Medical School ranking first and contributing far more than the remaining four (Figure 5A). Harvard Medical School mainly studies the inducing effect of gut microbiota on T cell differentiation and explores the potential of gut microbiota to treat immune-related cancers and autoimmune diseases through interaction with host immunity. The institute found that Fusobacterium may promote colon carcinogenesis. The increase in *Methanobrevibacter* and *Akkermansia* and the decrease in *Butyrimionas* in the gut microbiota of patients with multiple sclerosis are associated with changes in DC maturation, interferon signal transduction, and gene expression of nuclear factor (NF) -κB signal pathway in clinical trials (Mima et al., 2015; Jangi et al., 2016; Tanoue et al., 2019). The United States allots extensive spending for scientific research and has advanced medical research and clinical trial conditions. The same publication trend can also be documented in other diseases and related fields, such as research trends on sepsis and host immune response and links between gut microbiota and depression (Yao et al., 2020; Zhu et al., 2020). In addition, the United States started early in the research of gut microbiota and has the most frequently cited review (Table 1). The current review systematically summarized previous studies on the relationship between intestinal microbiota and the immune system, identified the challenges and prospects, and laid a foundation for further exploration of the complex mechanism between microbiota and host immunity (Hooper et al., 2012).

China accounted for the second most publications. The number of publications in China far exceeded those in the lower-ranking countries, but its citation frequency and H-index were not as high (Figure 3A). Several reasons account for the contradiction between the quantity and quality of Chinese publications. First, due to the strong support of the Chinese government and the implementation of relevant policies, China has continuously attached importance to research in natural science (Yuan et al., 2021). Second, China started late in this field and hence has no publication of relevant articles and no solid research foundation in other countries in the early stage. Therefore, China's citation frequency requires a long time to catch up with other countries. Finally, traditional Chinese herbs are a characteristic of traditional Chinese medicine; however, due to the complexity of their components, metabolites, and biological activities, the existing technical methods may encounter difficulties when applied in this research (Chen et al., 2021). Therefore, clarifying the complex mechanism of traditional Chinese medicine on gut microbiota should be prioritized by Chinese researchers. No Chinese article belonged to the top 10, (Table 1) and only one Chinese institution—Shanghai Jiao Tong University—was included in the top 10 list of contributions of different institutes (Figure 5A). This finding indicates that top Chinese institutions are needed to improve the international status of gut microbiota-related immune research. Shanghai Jiao Tong University used Chinese herbal formula to improve the structure of gut microbiota to alleviate the symptoms of type 2 diabetes. The formula was observed to enrich *F. prausnitzii* which is positively correlated with the functional homeostasis of B cells in real-time PCR (Xu et al., 2015). The concept of this clinical trial involves the unique features of Chinese traditional medicine. As an oral preparation, traditional Chinese medicine contains various components that might come in contact with gut microbiota after ingestion. This process includes the diversity and structural changes of microbiota and the occurrence of a variety of complex physiological mechanisms. Therefore, Chinese researchers must focus on the micro world of traditional Chinese medicine and explore the complex mechanism between traditional Chinese medicine and microbiota to publish high-quality and in-depth articles.

The national cooperative relationship network (Figure 4) indicates that the United States and China are the main parts of cooperative countries. The United States and China cooperate with most countries and have a complex cooperative relationship network. However, China's cooperation with other countries is far less close than that of the United States. China has just begun to pay attention to this field, and the quality of Chinese publications is not sufficiently high. Interestingly, we have found that most of the published studies in this field were from developed countries, whereas few studies were conducted from developing countries. Imbalances in economic development and different policies specified by different countries could cause the regional imbalance in the production of knowledge on the gut microbiome and host immune response research. Developed countries with high GDP have more funds to support research institutions and technological innovation, and researchers prefer high-quality scientific research platforms in these developed countries. In general, countries that cooperate with other countries rank high in publication output. The international collaborative capacity of a country can indirectly reflect the scientific productivity of a country. Taken together with previous bibliometric studies (Ilagan-Vega et al., 2022; Tantengco and Rojo, 2022), international collaboration may be served as one of the strongest predictors of scientific productivity. Therefore, all countries and institutes should strengthen international exchanges and cooperation.

Journal contributions analysis and journal citation analysis can provide a clear direction for researchers in writing and publishing articles. At present, the research in the field of intestinal microbiota and host immune response is relatively concentrated, and the secondary subjects in the top 10 journals mainly involve medicine, immunology, microbiology, and molecular biology (Figure 6A). Among which, the three journals' IF is >10, and the IF of the highest journal *Immunity* is 31.745. This finding indicates that researchers pay sufficient attention to the construction of journals in this field. *Frontiers in Immunology* is popular with researchers, and this may be related to the journal's broad reach and audience.

### Research focuses on gut microbiota and host immune response

The high-frequency keywords derived from bibliometric and visual analysis can be broadly classified into four main categories (Figure 7A): animal (red part), human (blue part), cell (yellow part), and disease (green part). “T cell” is at the center of the visualization plot and is closely linked to other categories of keywords and therefore plays an essential role in the study of gut microbiota and host immunity. Among the top 10 articles (Table 1), two are related to the effects of gut microbiota or microbiota metabolites on the differentiation of T cells. Atarashi et al. (2013) isolated Clostridia strains from human feces and colonized germ-free mice and found 17 strains of Clostridia after colonization. They also created a community that provides bacterial antigens and is enriched in transforming growth factor (TGF)-β, which enhances the abundance of CD4 ^+^ Foxp3 ^+^ Treg cells and induces an increase in anti-inflammatory factors including interleukin (IL)-10. In addition, the 17 strains were able to reduce symptoms in adult mouse models of colitis and allergic diarrhea, and three were devoid of prominent toxins and virulence factors, thus offering the possibility of further use of the isolated strains for human immune diseases. Arpaia et al. (2013) found that butyrate and propionate from SCFA produced by commensal bacteria increase the extrathymic differentiation of Treg cells. The Treg cells are one of the important factors for maintaining immune tolerance in the body, and commensal bacteria metabolites can mediate the communication of gut microbiota with the host immune system by affecting Treg cells and maintaining the balance between anti-inflammatory and pro-inflammatory mechanisms. All these findings suggest that Treg cells provide a bridge to regulatory communication between the gut microbiota and the host immune system.

As shown in Figure 7B, the keywords appeared from bottom to top as a function of time because exploring the complex mechanisms of the field is a step-by-step process. Researchers may start by noting that the occurrence and development of gastrointestinal diseases are closely related to the gut microbiota, through animal experiments to establish relevant animal models, to explore the association between microbiota and gastrointestinal diseases. In time, gut microbiota function will eventually be connected to systemic organs through metabolic, immune, and endocrine aspects and can affect human health and disease. Further exploration of mechanisms by which gut microbiota maintain health and cause disease is conducted based on animal experiments. However, human studies are still far behind animal models, and more rigorous experiments and theories are needed to provide feasibility for the application of animal experimental results to humans. In recent years, the major focus of human gut microbiota research is the relationship between age and gender in the microbiota (Yuan et al., 2021). Future high-quality clinical research directions may tilt toward personalized treatments with the gut microbiota.

Analysis of the occurrence frequency of the top 20 keywords by burst detection (Figure 6C) shows that in addition to the search keywords such as immune response and gut, “segmented filamentous bacteria (SFB)” and “commensal bacteria” have attracted considerable attention from peer researchers over the past decade, whereas “germ free” had the longest duration of hotness. SFB, as the first commensal bacteria to be discovered, are tightly attached to the intestinal epithelium of a variety of vertebrate and invertebrate species, and this tight adhesion to intestinal epithelial cells is a key cue for SFB-induced T helper (Th) 17 cells (Schnupf et al., 2013; Atarashi et al., 2015; Hedblom et al., 2018). Many complex signaling pathways have been implicated in SFB-induced Th17 cell generation, including MHCII antigen presentation by DC, secretion of serum amyloid A proteins (SAA1 and SAA2) from intestinal epithelial cells, and IL-22 expression in type 3 innate lymphoid cells (Goto et al., 2014; Sano et al., 2015; Lee et al., 2020). Notably, the expression of IL-17A, the main effector cytokine in the IL-17 subset, is restricted exclusively to the site of SFB attachment to the terminal ileum epithelium (Sano et al., 2015). IL-17A in the intestinal wall is not only involved in the regulation of intestinal wall inflammation but also has a close association with multiple organ diseases throughout the body, such as in the central nervous system (Beurel and Lowell, 2018), while Th17 cells and IL-17A can also reach peripheral sites and exacerbate autoimmune inflammation (Flannigan and Denning, 2018). SFB thus becomes an important loop in the study of immune system-related diseases.

“Probiotics” and “probiotic bacteria” simultaneously appear in the top 20 keywords (Figure 7C), illustrating that exogenous probiotics or intestinal beneficial bacteria are closely related to the host immune response. Although several clinical trials have demonstrated the effectiveness of probiotics in *H. pylori* eradication, reducing the incidence and severity of respiratory infections, reducing depression, preventing or treating atopic dermatitis, and reducing cardiovascular risk factors associated with cardiometabolic syndrome (Sniffen et al., 2018), equally high-quality studies with negative or opposite results for most of the above indications are conducted. Different ecological conditions in the host and gut microbiota might have led to different outcomes when different individuals were supplemented with the same probiotic preparation. Therefore, a personalized protocol must be applied in clinical research on probiotics and must involve the treated population and the disease targeted to tailor a unique probiotic preparation for each individual.

Researchers have focused their attention on SCFA, a metabolite of the gut microbiota (Figure 7C). SCFAs in the gut mainly include acetate, propionate, and butyrate, which are important energy sources for the gut microbiota and enterocytes with numerous immunomodulatory functions, particularly butyrate. SCFA concentration in the gut depends on the composition of the microbiota, the rate of SCFA metabolism, and the content of dietary fiber (Rooks and Garrett, 2016; Martin-Gallausiaux et al., 2021). SCFA acting as inhibitors of histone deacetylases (HDACs) can inhibit the NF-κB pathway and down-regulate the expression of the pro-inflammatory cytokine tumor necrosis factor -α to promote tolerogenic and anti-inflammatory cellular phenotypes, which is essential for maintaining immune homeostasis (Usami et al., 2008; Vinolo et al., 2011). In addition, SCFA can also act as ligands for G protein-coupled receptors (GPCRs) including GPR43, GPR41, and GPR109A, which can be expressed in immune cells and intestinal epithelial cells (Parada Venegas et al., 2019). Activated by SCFA, GPCRs can activate signaling cascades that control immune function to activate inflammasomes and subsequently induce IL-18 release, a pathway that can mediate protective immunity and tissue inflammation and is protective in murine models of inflammation (Kim et al., 2013; Macia et al., 2015). Trompette et al. (2014) reported that bone marrow hematopoiesis is altered after propionate treatment in mice, showing the enhanced synthesis of macrophage and DC precursors. Then DCs with high phagocytic capacity but impaired Th2 cell effector function are subsequently seeded in the lungs protecting the lungs from allergic inflammation. Erny et al. (2015) found that SCFA can regulate central nervous system tissue macrophage — microglia homeostasis and microglial damage can be corrected to some extent by the complex gut microbiota through experiments with germ-free mice. In clinical studies, consumption of relevant probiotics and dietary fibers can increase the relative concentrations of SCFA-producing probiotics and SCFA to ameliorate some diseases, such as type 2 diabetes (Zhao et al., 2018).

Two of the top 10 articles (Table 1) are about the role of gut microbiota modulating PD-1 for tumor therapy (Gopalakrishnan et al., 2018; Routy et al., 2018). It was found that patients with melanoma and a highly diverse and abundant gut microbiota of *Ruminococcaceae* and *Faecalibacterium* have enhanced systemic and antitumor immune responses mediated by increased antigen presentation and improved effector T cell function in the periphery and tumor microenvironment (Gopalakrishnan et al., 2018). Moreover, in murine experiments, *Akkermansia muciniphila* and *Enterococcus hirae* could induce DC to secrete IL-12, a Th1 cytokine involved in the immunogenicity of PD-1 blockade, and increase the efficacy of PD-1 blockade on tumor growth (Routy et al., 2018). These findings highlight the therapeutic potential of modulating the gut microbiome in patients receiving the immune checkpoint inhibitor (ICI) therapy, such that it may become feasible for the microbiome to manipulate the gut ecosystem to circumvent resistance to ICI. Research in the field of cancer has been a hotspot in medicine and biology at the frontier of science. The gut microbiota establishes a link to cancer through host immunity and influences tumor development and antitumor immunotherapy efficacy. Improving the efficacy of cancer immunotherapy can be considered in terms of microbial agents with immunostimulatory or immunosuppressive properties.

For the latest hotspot within all clusters, “COVID” appears the most recent (Supplemental Table 1). Since the outbreak of COVID-19, experts from various fields including molecular biology, medicine, nursing, immunology, and basic biology have come together to face this global life safety conundrum (Xia et al., 2021). In the realm of COVID-19, research on gut microbiota-immunity has also made some headway. The COVID-19 patients have an altered gut microbiota structure with a reduction in several commensals with immunomodulatory potentials, such as *F. prausnitzii, Eubacterium rectale*, and *Bifidobacteria*, and this perturbed composition is consistent with elevated concentrations of inflammatory cytokines and blood markers (Yeoh et al., 2021). Therefore, gut microbiome might be involved in the disease course of COVID-19 through cross-talk with the host immune response. In addition, the proposal of the gut-lung axis concept has enabled a close association between COVID-19 and the gut microbiota (Budden et al., 2017).

The emergence of each keyword in Figure 7, which represents the progress of the last decade in the field of gut microbiota and host immune response research and forms an integrated collaborative network: humans alter the diversity and structure of the indigenous gut microbiota and increase the production of beneficial metabolites by supplementation with corresponding probiotics and dietary fibers. These microbiota and metabolites ultimately affect human health and systemic diseases by interacting with the host immune system through complex mechanisms. In summary, research approaches in this area appear to have moved from traditional *in vitro* strain culture techniques to 16S rRNA sequencing and metagenomic sequencing, with research directions moving from the microbiota to microbial metabolites, such as SCFA, and research focuses moving from basic research to clinical applications. However, Figure 7B shows that clinically applied research started later than basic research, suggesting a lag in clinical research. There is a critical need to translate research findings into therapeutics for the tangible benefit of patients, a priority goal that researchers have been trying to break through.

## Limitation

This study searched the WOS database for publications in the field of gut microbiota and host immune response and obtained relatively objective search results. However, the advantages of this strategy, that dual-map overly analysis, were not highlighted in our study (You et al., 2021a). These issues would be focused to be addressed in subsequent studies. Owing to the exclusion of non-English publications, some key non-English studies might have been excluded from databases and analyses. In addition, only studies from the past decade were retrieved. The initial discovery process for the interaction between gut microbiota and host immune response might have been excluded. Future studies should expand the databases retrieved and the years of retrieval. Databases such as Medline, Scopus, or Google Scholar could be adopted and compared in further study and exploring 20 years or 30 years of research will enrich our results.

## Conclusion

This study comprehensively evaluated global research trends and hotspots in the field of gut microbiota and host immune responses through bibliometric and visual analysis. Future research directions were also predicted. A close collaboration between institutions has occurred in the last decade, and the number of published articles has increased rapidly since inception but decreased in the most recent years. The United States had the largest contribution in this field. China accounted for a considerable number of publications, but the quality and impact of papers must be further improved. The gut microbiota metabolite SCFA will continue to receive researcher attention in the future. However, breakthrough innovations in related experimental techniques are a big priority challenge in this field. In conclusion, the study of gut microbiota requires the participation of medical, biological, and other domain experts.

## Author contributions

ZN, SW, and YL collected and analyzed the data and wrote the manuscript. LZ and DZ designed the study and revised the manuscript. DX and CY provided the methodology. All authors read and approved the final manuscript. All authors contributed to the article and approved the submitted version.

## Funding

This work was supported by the National Natural Science Foundation of China (82004408) and Technology Innovation Action Plan of Shanghai Science and Technology Commission (No. 21Y21920500).

## Conflict of interest

The authors declare that the research was conducted in the absence of any commercial or financial relationships that could be construed as a potential conflict of interest.

## Publisher's note

All claims expressed in this article are solely those of the authors and do not necessarily represent those of their affiliated organizations, or those of the publisher, the editors and the reviewers. Any product that may be evaluated in this article, or claim that may be made by its manufacturer, is not guaranteed or endorsed by the publisher.
