# Supplementary material for: Mapping trends and hotspot regarding gut microbiota and host immune response: A bibliometric analysis of global research (2011–2021)
Source: Front Microbiol. 2022 Jul 22;13:932197. doi: 10.3389/fmicb.2022.932197 (PMC9361022; doi:10.3389/fmicb.2022.932197)
Supplement: Supplementary file 1 [file Table_1.DOCX]

**Supplemental Table 1. The analytic consequence of 213 keywords with at least 60 occurrence times.**

| **No.** | **Label** | **Cluster** | **Occurrences** | **Average appearing years  (AAY)** |
| --- | --- | --- | --- | --- |
| **1** | **absence** | **1** | **109** | **2017.0917** |
| **2** | **abundance** | **3** | **444** | **2019.0045** |
| **3** | **adaptive immune response** | **2** | **83** | **2016.3976** |
| **4** | **adaptive immune system** | **2** | **64** | **2016.3438** |
| **5** | **adaptive immunity** | **2** | **78** | **2016.8846** |
| **6** | **administration** | **1** | **313** | **2017.901** |
| **7** | **age** | **3** | **258** | **2017.6977** |
| **8** | **allergy** | **2** | **128** | **2017.1562** |
| **9** | **antigen** | **2** | **324** | **2017.0463** |
| **10** | **article** | **2** | **90** | **2018.3** |
| **11** | **association** | **3** | **279** | **2018.129** |
| **12** | **asthma** | **2** | **154** | **2017.6558** |
| **13** | **autoimmune disease** | **2** | **249** | **2017.5703** |
| **14** | **autoimmunity** | **2** | **203** | **2017.2906** |
| **15** | **b cell** | **1** | **204** | **2017.5392** |
| **16** | **bacterial translocation** | **1** | **97** | **2017.3608** |
| **17** | **bacteroide** | **3** | **82** | **2018.5366** |
| **18** | **bacteroidetes** | **3** | **81** | **2018.9012** |
| **19** | **beta** | **1** | **310** | **2017.7194** |
| **20** | **bifidobacterium** | **3** | **156** | **2017.7949** |
| **21** | **birth** | **2** | **91** | **2016.2747** |
| **22** | **blood** | **4** | **149** | **2017.5369** |
| **23** | **body** | **2** | **206** | **2017.7621** |
| **24** | **brain** | **2** | **93** | **2018.8172** |
| **25** | **butyrate** | **1** | **243** | **2018.5885** |
| **26** | **c57bl** | **1** | **101** | **2018.703** |
| **27** | **cancer** | **3** | **418** | **2019.2321** |
| **28** | **cancer immunotherapy** | **3** | **72** | **2019.4028** |
| **29** | **cause** | **2** | **111** | **2018.2072** |
| **30** | **cd4** | **4** | **370** | **2017.5811** |
| **31** | **central nervous system** | **2** | **63** | **2018.2698** |
| **32** | **child** | **4** | **146** | **2017.7808** |
| **33** | **cns** | **2** | **72** | **2018** |
| **34** | **colitis** | **1** | **658** | **2017.8875** |
| **35** | **colon** | **1** | **226** | **2018.0796** |
| **36** | **colorectal cancer** | **3** | **128** | **2019.0625** |
| **37** | **commensal** | **2** | **210** | **2016.8857** |
| **38** | **commensal bacterium** | **2** | **382** | **2016.2225** |
| **39** | **commensal microbiota** | **2** | **129** | **2016.845** |
| **40** | **concentration** | **1** | **200** | **2018.37** |
| **41** | **context** | **2** | **121** | **2018.2066** |
| **42** | **control group** | **3** | **97** | **2018.7629** |
| **43** | **correlation** | **3** | **150** | **2018.6733** |
| **44** | **covid** | **3** | **60** | **2020.8833** |
| **45** | **crc** | **3** | **115** | **2019.3478** |
| **46** | **crohn** | **4** | **106** | **2017.0189** |
| **47** | **day** | **1** | **375** | **2018.5653** |
| **48** | **dcs** | **1** | **234** | **2016.5983** |
| **49** | **decrease** | **1** | **163** | **2018.3681** |
| **50** | **dendritic cell** | **1** | **255** | **2016.7843** |
| **51** | **depletion** | **1** | **173** | **2017.1272** |
| **52** | **diabete** | **4** | **78** | **2017.4744** |
| **53** | **diabetes** | **4** | **155** | **2017.5484** |
| **54** | **differentiation** | **1** | **253** | **2017.249** |
| **55** | **disease** | **2** | **2261** | **2017.7762** |
| **56** | **disorder** | **2** | **477** | **2018.0021** |
| **57** | **diversity** | **3** | **490** | **2018.6122** |
| **58** | **dss** | **1** | **179** | **2018.8045** |
| **59** | **e coli** | **1** | **104** | **2017.3942** |
| **60** | **efficacy** | **3** | **272** | **2019.1654** |
| **61** | **environmental factor** | **2** | **128** | **2017.125** |
| **62** | **epithelial cell** | **2** | **146** | **2016.6575** |
| **63** | **escherichia coli** | **1** | **98** | **2017.5102** |
| **64** | **evidence** | **2** | **525** | **2018.0971** |
| **65** | **expansion** | **4** | **132** | **2017.6591** |
| **66** | **expression** | **1** | **1159** | **2017.7601** |
| **67** | **fecal microbiota** | **3** | **78** | **2018.2821** |
| **68** | **fecal microbiota transplantation** | **3** | **76** | **2019.4211** |
| **69** | **fecal sample** | **3** | **84** | **2018.4048** |
| **70** | **fece** | **3** | **108** | **2018.8796** |
| **71** | **firmicute** | **3** | **94** | **2018.234** |
| **72** | **flow cytometry** | **4** | **121** | **2018.3967** |
| **73** | **fmt** | **3** | **159** | **2019.3208** |
| **74** | **focus** | **2** | **98** | **2018.0816** |
| **75** | **foxp3** | **4** | **102** | **2016.7059** |
| **76** | **frequency** | **4** | **161** | **2017.7143** |
| **77** | **gastrointestinal tract** | **2** | **214** | **2017.3271** |
| **78** | **generation** | **1** | **108** | **2016.7315** |
| **79** | **group** | **3** | **818** | **2018.6968** |
| **80** | **gut brain axis** | **2** | **91** | **2019.2637** |
| **81** | **gut homeostasis** | **2** | **87** | **2017.4368** |
| **82** | **gut microbiome** | **3** | **905** | **2018.9779** |
| **83** | **health** | **2** | **457** | **2017.9037** |
| **84** | **healthy control** | **3** | **110** | **2018.8727** |
| **85** | **hfd** | **1** | **116** | **2017.9397** |
| **86** | **high fat diet** | **1** | **70** | **2018.2429** |
| **87** | **hiv** | **4** | **309** | **2017.2136** |
| **88** | **homeostasis** | **2** | **658** | **2017.4833** |
| **89** | **host** | **2** | **732** | **2016.9795** |
| **90** | **host immune system** | **2** | **135** | **2017.4444** |
| **91** | **host immunity** | **2** | **104** | **2017.4519** |
| **92** | **human health** | **2** | **82** | **2017.6098** |
| **93** | **iec** | **1** | **66** | **2016.5455** |
| **94** | **ifn gamma** | **1** | **159** | **2017.4717** |
| **95** | **iga** | **1** | **343** | **2017.5073** |
| **96** | **ileum** | **1** | **80** | **2018.0625** |
| **97** | **immune activation** | **4** | **130** | **2017.4462** |
| **98** | **immune checkpoint inhibitor** | **3** | **67** | **2019.8806** |
| **99** | **immune system** | **2** | **890** | **2017.1697** |
| **100** | **immunoglobulin** | **1** | **103** | **2017.5534** |
| **101** | **immunotherapy** | **3** | **184** | **2019.8641** |
| **102** | **implication** | **2** | **130** | **2017.8615** |
| **103** | **importance** | **2** | **146** | **2017.6986** |
| **104** | **incidence** | **4** | **98** | **2017.602** |
| **105** | **increase** | **1** | **367** | **2018.2643** |
| **106** | **individual** | **4** | **290** | **2017.4621** |
| **107** | **induction** | **1** | **304** | **2016.9704** |
| **108** | **infant** | **3** | **160** | **2016.8812** |
| **109** | **inflammatory cytokine** | **1** | **150** | **2018.3733** |
| **110** | **inflammatory disease** | **2** | **171** | **2017.1813** |
| **111** | **influence** | **2** | **220** | **2017.4136** |
| **112** | **inhibition** | **1** | **109** | **2018.1101** |
| **113** | **insight** | **2** | **211** | **2017.9716** |
| **114** | **interaction** | **2** | **867** | **2017.4014** |
| **115** | **interleukin** | **1** | **178** | **2017.3708** |
| **116** | **interplay** | **2** | **155** | **2018.3548** |
| **117** | **intestinal epithelial cell** | **1** | **113** | **2016.8496** |
| **118** | **intestinal homeostasis** | **2** | **152** | **2017.2763** |
| **119** | **knowledge** | **2** | **144** | **2017.9167** |
| **120** | **lactobacillus** | **3** | **95** | **2018.3053** |
| **121** | **level** | **1** | **1198** | **2018.2287** |
| **122** | **life** | **2** | **177** | **2017.6836** |
| **123** | **link** | **2** | **140** | **2018.2** |
| **124** | **lipopolysaccharide** | **1** | **138** | **2018.0725** |
| **125** | **lps** | **1** | **267** | **2017.8764** |
| **126** | **macrophage** | **1** | **549** | **2017.8871** |
| **127** | **maintenance** | **2** | **157** | **2016.8153** |
| **128** | **mait cell** | **4** | **80** | **2018.575** |
| **129** | **mesenteric lymph node** | **1** | **104** | **2017.7308** |
| **130** | **metabolic disease** | **2** | **68** | **2018.1471** |
| **131** | **metabolite** | **2** | **603** | **2018.9635** |
| **132** | **mice** | **1** | **211** | **2018.3318** |
| **133** | **microbe** | **2** | **673** | **2017.3997** |
| **134** | **microbiome** | **2** | **1194** | **2018.4732** |
| **135** | **microorganism** | **2** | **306** | **2017.1536** |
| **136** | **mln** | **1** | **62** | **2017.871** |
| **137** | **molecular mechanism** | **2** | **83** | **2017.4096** |
| **138** | **monocyte** | **1** | **130** | **2016.7923** |
| **139** | **month** | **3** | **93** | **2017.5914** |
| **140** | **mouse** | **1** | **3242** | **2017.946** |
| **141** | **mucosal** | **4** | **101** | **2016.8911** |
| **142** | **mucosal immune system** | **2** | **96** | **2016.5312** |
| **143** | **multiple sclerosis** | **2** | **110** | **2018.3091** |
| **144** | **nf kappa b** | **1** | **94** | **2018.5** |
| **145** | **nutrient** | **2** | **94** | **2017.5319** |
| **146** | **obesity** | **1** | **224** | **2018.1384** |
| **147** | **onset** | **4** | **146** | **2017.7534** |
| **148** | **oral administration** | **1** | **92** | **2017.7609** |
| **149** | **outcome** | **3** | **253** | **2018.7115** |
| **150** | **patch** | **1** | **81** | **2016.6543** |
| **151** | **pathogen** | **2** | **435** | **2016.8943** |
| **152** | **pathogenesis** | **2** | **474** | **2018** |
| **153** | **patient** | **3** | **1617** | **2018.6463** |
| **154** | **percentage** | **4** | **75** | **2018.4533** |
| **155** | **peyer** | **1** | **93** | **2016.5591** |
| **156** | **pig** | **1** | **96** | **2017.3542** |
| **157** | **prebiotic** | **2** | **118** | **2017.2034** |
| **158** | **present study** | **1** | **95** | **2018.5579** |
| **159** | **probiotic bacterium** | **1** | **80** | **2016.4** |
| **160** | **proliferation** | **1** | **167** | **2017.3413** |
| **161** | **proportion** | **4** | **157** | **2018.1656** |
| **162** | **protective effect** | **1** | **105** | **2018.4571** |
| **163** | **proteobacteria** | **3** | **74** | **2018.6892** |
| **164** | **psoriasis** | **3** | **70** | **2019.5286** |
| **165** | **rat** | **1** | **226** | **2017.7345** |
| **166** | **ratio** | **3** | **207** | **2018.5556** |
| **167** | **recent finding** | **2** | **65** | **2017.4308** |
| **168** | **recent study** | **2** | **129** | **2017.9302** |
| **169** | **reduction** | **1** | **190** | **2018.0421** |
| **170** | **relationship** | **2** | **396** | **2017.803** |
| **171** | **relative abundance** | **3** | **167** | **2019.1198** |
| **172** | **research** | **2** | **205** | **2018.4927** |
| **173** | **review** | **2** | **616** | **2018.0455** |
| **174** | **rheumatoid arthritis** | **2** | **66** | **2017.9242** |
| **175** | **risk** | **2** | **225** | **2018.1022** |
| **176** | **scfa** | **1** | **184** | **2019.0924** |
| **177** | **scfas** | **1** | **172** | **2018.6221** |
| **178** | **secretion** | **1** | **206** | **2017.8058** |
| **179** | **sepsis** | **3** | **116** | **2018.7069** |
| **180** | **serum** | **1** | **110** | **2018.5273** |
| **181** | **sfb** | **1** | **115** | **2016.5217** |
| **182** | **significant difference** | **3** | **60** | **2018.7333** |
| **183** | **skin** | **2** | **80** | **2016.925** |
| **184** | **small intestine** | **1** | **117** | **2017.2222** |
| **185** | **stimulation** | **1** | **173** | **2017.1098** |
| **186** | **strain** | **1** | **563** | **2017.1243** |
| **187** | **subject** | **3** | **182** | **2017.2912** |
| **188** | **subset** | **4** | **240** | **2016.9583** |
| **189** | **supplementation** | **1** | **250** | **2018.968** |
| **190** | **t cell** | **4** | **1130** | **2017.5239** |
| **191** | **t1d** | **4** | **82** | **2017.7561** |
| **192** | **therapy** | **3** | **450** | **2018.6067** |
| **193** | **tlr** | **1** | **169** | **2016.568** |
| **194** | **tlr4** | **1** | **81** | **2016.8272** |
| **195** | **tnf alpha** | **1** | **197** | **2018.1421** |
| **196** | **tolerance** | **2** | **296** | **2016.8953** |
| **197** | **toll** | **1** | **188** | **2016.6702** |
| **198** | **translocation** | **1** | **211** | **2017.7441** |
| **199** | **treg** | **4** | **194** | **2017.6546** |
| **200** | **treg cell** | **4** | **130** | **2017.4308** |
| **201** | **tregs** | **4** | **73** | **2017.137** |
| **202** | **tumor** | **3** | **155** | **2019.7548** |
| **203** | **turn** | **2** | **75** | **2018.1733** |
| **204** | **type** | **4** | **441** | **2017.5714** |
| **205** | **ulcerative colitis** | **1** | **115** | **2018.0609** |
| **206** | **understanding** | **2** | **304** | **2017.4901** |
| **207** | **variety** | **2** | **87** | **2017.2529** |
| **208** | **virus** | **2** | **170** | **2018.1118** |
| **209** | **vitro** | **1** | **183** | **2017.6503** |
| **210** | **vivo** | **1** | **64** | **2017.4375** |
| **211** | **week** | **1** | **254** | **2018.3543** |
| **212** | **wild type** | **1** | **74** | **2017.4054** |
| **213** | **year** | **3** | **184** | **2017.3315** |
